# A Reliable and Rapid Language Tool for the Diagnosis, Classification, and Follow-Up of Primary Progressive Aphasia Variants

**DOI:** 10.3389/fneur.2020.571657

**Published:** 2021-01-05

**Authors:** Stéphane Epelbaum, Yasmina Michel Saade, Constance Flamand Roze, Emmanuel Roze, Sophie Ferrieux, Céline Arbizu, Marie Nogues, Carole Azuar, Bruno Dubois, Sophie Tezenas du Montcel, Marc Teichmann

**Affiliations:** ^1^Department of Neurology, National Reference Center for “PPA and rare dementias”, Institute for Memory and Alzheimer's Disease, Pitié Salpêtrière Hospital, AP-HP, Paris, France; ^2^Institut du Cerveau, ICM, INSERM U 1127, CNRS UMR 7225, Sorbonne Université, Paris, France; ^3^Inria, Aramis-project team, ‘APHP-INRIA collaboration’, Paris, France; ^4^Centre Hospitalier Sud-Francilien, Université Paris Sud, Corbeil-Essonnes, Service de Neurologie et Unité Neurovasculaire, Corbeil-Essonnes, France; ^5^Department of Neurology, Pitié Salpêtrière Hospital, AP-HP, Paris, France; ^6^Sorbonne Université, INSERM, Institut Pierre Louis d'Épidémiologie et de Santé Publique, AP-HP, Hôpitaux Universitaires Pitié Salpêtrière - Charles Foix, Paris, France

**Keywords:** primary progressive aphasia, Alzheiemer's disease, language, test, diagnosis

## Abstract

**Background:** Primary progressive aphasias (PPA) have been investigated by clinical, therapeutic, and fundamental research but examiner-consistent language tests for reliable reproducible diagnosis and follow-up are lacking.

**Methods:** We developed and evaluated a rapid language test for PPA (“PARIS”) assessing its inter-examiner consistency, its power to detect and classify PPA, and its capacity to identify language decline after a follow-up of 9 months. To explore the reliability and specificity/sensitivity of the test it was applied to PPA patients (*N* = 36), typical amnesic Alzheimer's disease (AD) patients (*N* = 24) and healthy controls (*N* = 35), while comparing it to two rapid examiner-consistent language tests used in stroke-induced aphasia (“LAST”, “ART”).

**Results:** The application duration of the “PARIS” was ~10 min and its inter-rater consistency was of 88%. The three tests distinguished healthy controls from AD and PPA patients but only the “PARIS” reliably separated PPA from AD and allowed for classifying the two most frequent PPA variants: semantic and logopenic PPA. Compared to the “LAST” and “ART,” the “PARIS” also had the highest sensitivity for detecting language decline.

**Conclusions:** The “PARIS” is an efficient, rapid, and highly examiner-consistent language test for the diagnosis, classification, and follow-up of frequent PPA variants. It might also be a valuable tool for providing end-points in future therapeutic trials on PPA and other neurodegenerative diseases affecting language processing.

## Introduction

Primary progressive aphasias (PPA) are neurodegenerative diseases characterized by isolated or highly predominant language impairment. They include three main variants: logopenic (lv-PPA), semantic (sv-PPA) and non-fluent/agrammatic PPA (nfv-PPA) (1). A growing number of clinical and fundamental investigations have explored PPA but the initial diagnosis, determining the inclusion of patients in such investigations, depends on the expertise of specialized neurologists whereas reliable, rapid and examiner-consistent diagnostic tests are lacking. Likewise, there are no tests assessing language decline during the follow-up of PPA patients, and thus no reliable evaluation tools of potential efficacy of therapeutic PPA trials. To address some of these issues several tests have been developed: the *Progressive Aphasia Language Scale* (“PALS”) (2), the *Progressive Aphasia Screening Scale* (“PASS”) (3) and the *Sydney Language Battery* (“SYDBAT”) (4), as well as the *Screening for Aphasia in NeuroDegeneration Battery (“SAND”)* battery (5, 6). However, the use and reliability of these tests are limited by time-consuming administration and/or subjective biases related to multi-point non-binary rating scales. It is also unknown whether these tests are sensitive to language decline during the disease course and whether they are able to differentiate PPA from the most common neurodegenerative condition, namely typical Alzheimer's disease (AD). On the other hand, two rapid language tests with excellent inter-examiner consistency have been developed and validated in post-stroke aphasia: the *Language Screening Test* (“LAST”) (7) and the *Aphasia Rapid Test* (“ART”) (8). These two tests might represent valuable tools in PPA but they were not designed for degenerative conditions requiring more specifically the detection, classification and follow-up of PPA patients. To address these open issues, we developed a rapid language test with a binary rating scale, designed for PPA (*Progressive Aphasia RatIng Scale* “PARIS”), while assessing its power for diagnosis/classification of PPA and its sensitivity to language decline, while comparing it with the two rapid and inter-rater consistent language tests: the “LAST” and “ART.” To evaluate more specifically its capacity to diagnose and differentiate PPA from other neurodegenerative conditions with aphasia features we applied the test both to PPA variants and typical amnesic AD.

## Methods

### Participants

Participants were consecutively recruited at the Institute for Memory and Alzheimer's Disease and the National Reference Center for PPA of the Pitié Salpêtrière Hospital, Paris, France. We included 36 patients with PPA (13 sv-PPA, 20 lv-PPA, 3 nfv-PPA) who were diagnosed and classified according to the current international diagnosis criteria (1). The identification of only 3 nfv-PPA patients in our consecutive patient series reflects the fact that genuine nfv-PPA is a rare phenotype when excluding patients with exclusively motor speech disorders who do not correspond to aphasia patients but rather to the syndromic framework of “primary progressive apraxia of speech” (9, 10). The procedure of PPA variant classification/diagnosis was performed for all patients before applying, in a second step, the PARIS, LAST and ART. This procedure was based on the clinical gold standard stipulating that diagnoses/classifications should be made by an expert neurologist in the field of PPA (2) according to the international diagnosis criteria (1). The expert neurologist of this study (M.T.) based diagnosis on his longstanding experience in the field and he used the results of the “General cognitive/language assessment” (see below) to strengthen/refine his PPA classification. Regarding nfv-PPA, the “General cognitive/language assessment” comprised several tests allowing for the detection of syntactic and articulation abilities during speech/language production. Speech therapists carefully analyzed the speech output in all these tests regarding articulatory/motor speech, syntactic, and phonological performance. Patients who had isolated dysarthria or apraxia of speech were not classified as PPA and were not included in the study. Patients with syntactic disorders +/– disorders of phonological encoding were classified as nfv-PPA. We also included 24 patients with typical amnesic AD diagnosed according to the most recent diagnostic research criteria of the International Working Group (IWG) stating that typical AD is defined by both an amnesic syndrome of the hippocampal type and a positive cerebrospinal fluid (CSF) biomarker profile (11). Furthermore, we included 35 healthy controls recruited via an announcement that healthy volunteers are needed for a research program. They mostly were spouses or friends of the patients. Controls, PPA and AD patients had similar characteristics regarding age and years of education (Mann-Whitney tests: all *p* > 0.05). All participants were native French speakers. Patients did not have other neurological disease than PPA or AD. Healthy controls had *Mini Mental State Examination* (MMSE) (12) scores > 27 and *Frontal Assessment Battery* (FAB) (13) scores > 15, reflecting normal cognitive functioning. All participants signed informed consent and the study was approved by the local Ethical committee. Demographic data of the participants are illustrated in Table 1.

**Table 1:** Demographic data of healthy controls, PPA and AD patients (mean and 95% Confidence Intervals).

	**Controls**	**PPA**				**AD**
		**all PPA**	**lv-PPA**	**sv-PPA**	**nfv-PPA**	
Number of subjects	35	36	20	13	3	24
Sex (women/men)	20/15	18/18	9/11	7/6	2/1	12/12
Age (years)	68.3 (65.6–71.1)	69 (65–74.5)	70 (66.5–73.1)	69.8 (65.4–74.3)	68.9 (59.7–78.2)	70 (66.7–73.3)
Handedness (right/left)	34/1	35/1	19/1	13/0	3/0	23/1
Years of education	14 (12-17)	15.5 (11.5–18)	15 (13.3–16.7)	14.4 (12.2–16.6)	14.7 (10.2–19.1)	13 (9-15)
Symptom duration (years)	NA	3 (2–4.5)	5.1 (4.2–5.9)	4 (2.9–5.2)	3.3 (1–5.7)	4 (3-7)

### General Cognitive/Language Assessment

The general cognitive assessment included the MMSE and the FAB. Episodic memory capacities were assessed with the *Free and Cued Selective Reminding Test* (FCSRT) as recommended by the IWG, (14, 15) for which an amnesic syndrome of the hippocampal type is defined by a total recall < 40/48, and/or a free recall < 17/48 (16). The FCSRT was only applied to AD because language impairment in PPA necessarily biases the results of this verbal memory test. The language assessment was composed of a picture naming test (D080) (17) and the Boston Diagnostic Aphasia Evaluation (BDAE) (18). The BDAE included an evaluation of aphasia severity taking into account spontaneous speech and the description of the “cookies theft picture,” a sentence repetition task, and a single-word comprehension task requiring pointing to pictures upon auditory word presentation. We also applied a verbal fluency test comprising phonemic and category fluency (19). Cognitive/language scores are summarized in Table 2.

**Table 2:** Cognitive/language scores of healthy controls, PPA and AD patients (mean and 95% Confidence Intervals).

**Tests**	**Controls**	**PPA**				**AD**	Comparisons^*^
		**all PPA**	**lv-PPA**	**sv-PPA**	**nfv-PPA**		
MMSE (/30)	28.1 (27–29.2)	22 (19-25)	20.2 (18.8–21.6)	23.9 (22.1–25.8)	24 (19.4–28.5)	19.8 (18.4–21.2)	Controls>all patient groups
FAB (/18)	16.6 (15.7–17.5)	12.1 (10.3–14.7)	11 (9.8–12.2)	13 (11.4–14.6)	12 (8.2–15.8)	12.7 (11.6–13.8)	Controls>all patients groups
FCSRT FR (/48)	NA	NA	NA	NA	NA	10.8 (7.8–12.2)	Normal threshold ≥ 17
FCSRT TR (/48)	NA	NA	NA	NA	NA	28.1 (26.5–29.9)	Normal threshold ≥ 40
BDAE – aphasia severity scale (/5)	NA	3 (2.3–3.7)	2.8 (2.3–3.5)	3.1 (2.7–3.7)	3.2 (2.8–3.4)	4 (3.2–4.7)	Equal for all patient groups
BDAE – single-word comprehension (/72)	NA	67.8 (65.5–71.6)	69.6 (67.5–71.5)	61.5 (60–63)	71 (70–72)	69 (68.5–70.5)	lv-PPA, nfv-PPA, AD>sv-PPA
BDAE – sentence repetition (/16)	NA	11.3 (8.5–12.2)	10.8 (8-13)	15 (14-16)	10 (9-11)	12.2 (12–14.2)	sv-PPA>lv-PPA,nfv-PPA,AD
Category fluency	NA	7.4 (6.5–8)	7.8 (7-8)	7 (5-8)	6 (5-7)	8.2 (6.7–10)	equal for all patient groups
Phonemic fluency	NA	7.3 (5.5–9)	7 (4-9)	7 (6-9)	9 (7-10)	16.2 (10–20.7)	AD>all PPA groups
DO80 (/80)	NA	61.4 (59–67.2)	57.8 (57–62)	44 (41–48)	77 (76–78)	70.2 (65.5–76.2)	nfv-PPA>AD>lv-PPA>sv-PPA
PARIS (/55)	51.7 (50.2–53.2)	40.5 (38.7–42.4)	39.6 (37.7–41.6)	41.8 (39.4–44.3)	41 (35.9–46.1)	44.8 (43–46.6)	Controls>AD>all PPA groups
LAST (/15)	14.9 (14.5–15)	13.1 (12.5–13.6)	12.8 (12.3–13.4)	13.2 (12.5–13.8)	14 (12.6–15.3)	14 (13.6–14.5)	Controls>all patient groups
ART (/26)	0.4 (0–1)	3.8 (3.1–4.6)	4.1 (3.4–4.9)	3.1 (2.2–4)	4.7 (2.8–6.5)	2.8 (2.1–3.4)	Controls>all patient groups

*FCSRT FR, Free and cued selective reminding test |Free Recall,” FCSRT TR, Free and cued selective reminding test “Total Recall”*.

* *Kruskal-Wallis with post-hoc Dwass-Steel-Critchlow-Fligner for pair-wise comparisons*.

*Only significant differences are shown (“>” indicates significantly better performances). NA, not applicable*.

### The Three Rapid Language Tests

#### Progressive Aphasia Rating Scale (PARIS)

We designed the test to rapidly and reproductively assess language in neurodegenerative conditions, and especially in PPA. It comprises ten subtests evaluating different language domains including lexical, semantic, phonological, morpho-syntactic and verbal short-term memory capacities. To ensure the sensitivity of the test the different items composing each subtest were constructed to provide increasing complexity in terms of linguistic parameters such as lexical frequency using the LEXIQUE 2 database (20), number of words and phonemic cluster complexity. Item familiarity (semantic frequency), which is generally correlated with lexical frequency, was not used for selecting the stimuli of the PARIS. Likewise, the parameter of “semantic category” was not used because it does not provide the possibility to construct a continuous complexity graduation. Scoring for each item was binary (correct = 1 point/incorrect = 0 points), except for the category and phonemic fluency subtests. For both fluency subtests the scores were semi-quantitative: 0–3 words: 0 points, 4–6 words: 1 point, 7–10 words: 2 points, 11–14 words: 3 points, >15 words: 4 points. The maximal score of the PARIS was 55/55. The subtests of the PARIS, their targeted language domains, and their potential sensitivity regarding PPA variants are illustrated in Table 3. The PARIS is shown in the Supplementary Figure 1. It should be noted that the sub-tests did not assess specifically particular language components, which would require implicit processing task that are not suitable for a rapid language assessment in clinical practice.

**Table 3:** Subtests of the PARIS.

**Subtests (number of items)**	**Targeted language domain**	**Targeted PPA variant**
Picture naming (8)	Semantic, lexical	sv-PPA, lv-PPA
Picture designation [single-word comprehension] (8)	Semantic	sv-PPA
Word repetition (8)	Phonological encoding	nfv-PPA, lv-PPA
Sentence repetition (5)	Verbal short-term memory	lv-PPA
Irregular word reading (4)	Semantic	sv-PPA
Irregular word writing (4)	Semantic	sv-PPA
Verb conjugation (6)	Morpho-syntactic	nfv-PPA
Phonemic fluency (P/1 minute)	Lexical access	lv-PPA
Category fluency (animals/1 minute).	Lexical access, semantic	sv-PPA, lv-PPA
Oro-facial praxis (4)	Proxy of motor speech performance	nfv-PPA

#### Language Screening Test (LAST)

The LAST has been extensively described and explored in the context of post-stroke aphasia (7). Briefly, it consists in a two-part test designed to assess language production and perception. It comprises 5 subtests evaluating picture naming (/5), word and sentence repetition (/2), counting from one to ten (/1), single-word comprehension (/5), and simple and complex order execution (/3). The maximal score of the LAST is 15/15. The LAST does not account for linguistic parameters such as item frequency.

#### Aphasia Rapid Test (ART)

The ART evaluates both language and dysarthria and has been evaluated in the context of post-stroke aphasia (8). Briefly, it consists in a two-part test designed to assess language production and perception. Its score is calculated depending on the result in different subtests which are ranked as normal (score “0”) or abnormal (score > “0”) depending on the severity of impairment (poorest performance = “4”). The subtests assess simple and complex order execution, word and sentence repetition, dysarthria, picture naming, and category fluency. The scoring is inversed in comparison to the two former tests (maximal score 0/26, minimal score 26/26). The ART does not account for linguistic parameters such as item frequency.

### Test Procedure

The PARIS, LAST, and ART were applied exclusively to patients with anteriorly established PPA variant or AD diagnosis. The application order of the three tests was counterbalanced within each patient population and within the healthy controls. Rating of performance for each participant was performed by two independent examiners who were non-experts in PPA or AD, and who were blind to the clinical diagnosis. The examiners were neurology residents and third-year medical students who were all fluent in French. To evaluate the sensitivity of the tests to detect language decline they were applied twice: at the initial test session (baseline) and at the follow-up session 9 (± 1.2) months after baseline.

### CSF Biomarker Analyses

CSF analyses were performed at the biochemistry department of the Pitié-Salpêtrière Hospital for all patients of the AD group to ensure accurate diagnosis. They were not performed for PPA patients given that CSF biomarker data are not necessary nor recommended for accurate PPA variant diagnosis. The analyses included the quantification of total tau protein (T-tau), tau protein phosphorylated at threonine 181 (p-tau_181_) and amyloid-β peptide 1-42 (Aβ_1−42_). CSF samples were centrifuged for 10 min at 3,500 rpm at 4°C in order to remove cells, then aliquoted to 0.4 ml samples in polypropylene tubes and stored at −80°C until analysis. Biomarker concentrations of T-tau, p-tau_181_ and Aβ_1−42_ were analyzed in duplicate using the double antibody sandwich ELISA method (Fujirebio®). We also calculated ratios from single biomarkers including T-tau/Aβ_1−42_ and p-tau_181_/Aβ_1−42_. The ratio cut-off indicative of AD was set at p-tau_181_/Aβ_1−42_ > 0.11 based on studies with post-mortem verification of AD diagnosis (21, 22), and on a large longitudinal monocenter cohort (23). This approach was used to provide robust cut-offs validated by neuropathological examinations.

### Statistical Analyses

As the scoring procedure of the maximum scores of the PARIS and LAST differed from the ART we converted results of each test into percentages of accuracy. Internal validity of the PARIS was analyzed by assessing its reliability through Cronbach's alpha computation and inter-examiner consistency was assessed by computing Cohen's kappa for each pair of examiners. To assess the capacity of the PARIS, LAST, and ART to discriminate between healthy controls, PPA and AD patients we used Kruskal-Wallis tests and *post-hoc* Dwass-Steel-Critchlow-Fligner tests for pair-wise comparisons. To determine the best classifying subtests of the PARIS, LAST and ART for distinguishing the two most frequent PPA variants, i.e., sv-PPA and lv-PPA (24), we applied Wilcoxon tests and Bonferroni correction for multiple comparisons. Patients with nfv-PPA were excluded from these analyses because of their small number (*N* = 3). We also evaluate the external validity of the PARIS although there is no standard aphasia battery designed to diagnose PPA. In this context we used two validated and well-known standard aphasia batteries/tests, the BDAE comprising multiple subtests and the DO80 picture naming test, while calculating Pearson's correlation coefficients for the comparison between PARIS subtest scores and corresponding subtest scores of the BDAE (sentence repetition, single-word comprehension) and scores of the DO80. We furthermore calculated Pearson's correlation coefficients for the comparison between PARIS, LAST, and ART scores. Discriminant validity of the PARIS was assessed by comparing these correlation coefficients to those of the comparisons between the PARIS and the MMSE and FAB.

Finally, to determine specificity and sensitivity values of the PARIS, as well as of the LAST and ART, we generated Receiver Operating Characteristic (ROC) curves for each test and all group comparisons (PPA vs. healthy controls, AD vs. healthy controls, PPA vs. AD). The optimal cut-off values of comparisons between healthy controls and PPA or AD were calculated by determining the score for each test that maximized the Youden's index (sensitivity + specificity − 1). For the comparison between PPA and AD we fixed a cut-off of at least 50% of sensitivity.

Regarding the follow-up assessment, sensitivity to language decline between baseline and the test session at 9 months was assessed using *standardized response mean*s (SRM = mean decline/standard deviation of decline) (25).

## Results

The application duration of the PARIS was 10.43 +/– 0.46 min. This duration was assessed in the first 20 patients who underwent the PARIS, notably to assess the “rapid” feasibility of the test. However, the application duration of the PARIS was not assessed in the follow-up visit, which could have provided an indicator reflecting the influence of aphasia severity on the application duration of the PPA. Application durations for the LAST and ART were not recorded in this study but two previous investigations indicate that they are ~2 min for the LAST and ~3 min for the ART (7, 8).

### Rapid Language Tests (Baseline)

Cronbach's alpha was 0.74 demonstrating good internal consistency. Cohen's Kappa scores' mean was of 0.88 indicating an excellent inter-examiner consistency for the PARIS. The application duration of the PARIS was <11 min for patients.

**Figure 1:**
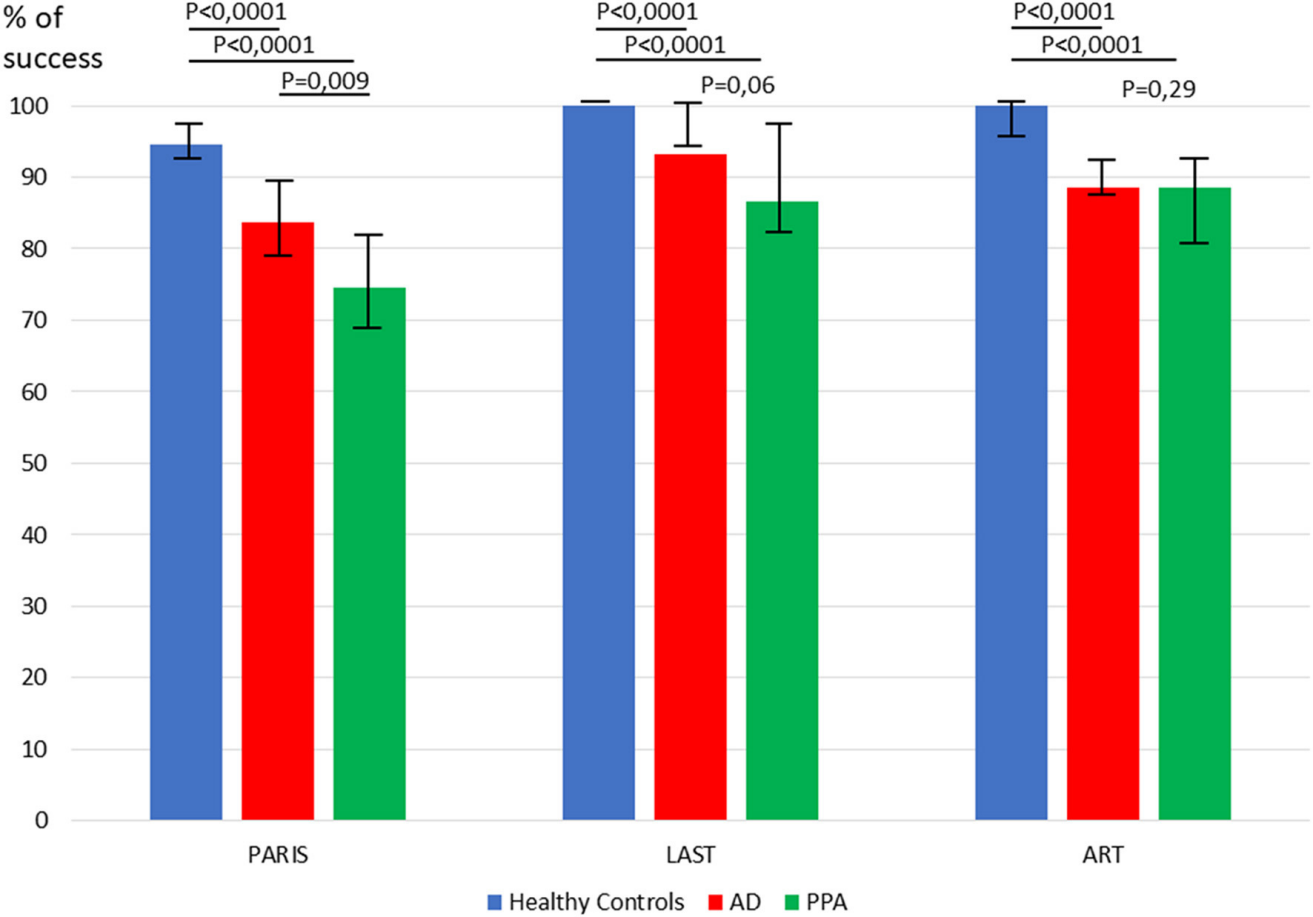
Comparison of healthy controls, PPA and AD patients for performances in the PARIS, LAST, and ART.

PARIS, LAST and ART reliably distinguished healthy controls and patients, but only PARIS scores were statistically different between AD and PPA (*p* = 0.009, see Figure 1). To evaluate the power to classify PPA variants we analyzed the different subtests of the PARIS. Scores of (i) the picture designation subtest (single-word comprehension) and (ii) the sentence repetition subtest, reliably distinguished lv-PPA and sv-PPA (*p* = 0.0001 and 0.0014, respectively (see Supplementary Table 1). A score of the sentence repetition subtest < 3/5 identified correctly 17/20 (85%) of lv-PPA patients, and a score of the picture designation subtest ≤ 7/8 identified correctly 10/13 (77%) of sv-PPA patients. The LAST and ART could not separate lv-PPA from sv-PPA.

Regarding external validity of the PARIS we found significant correlations, considering all participants, between its picture naming subtest and scores of the DO80 (Rho = 0.77, *p* < 0.0001), the subtests of “sentence repetition” of the PARIS and the BDAE (Rho 0.54, *p* < 0.0001), and the subtests assessing single-word comprehension of the PARIS and the BDAE (Rho 0.53, *p* < 0.0001). Furthermore, considering all participants, PARIS, LAST, and ART scores were significantly correlated (PARIS / LAST: Rho = 0.75, *p* < 0.0001, PARIS / ART: Rho = 0.84, *p* < 0.0001), ART / LAST (Rho = 0.71, *p* < 0.0001). Comparatively, the correlations between the PARIS and the FAB or MMSE scores were numerically lower (Rho = 0.33 and Rho = 0.41, respectively) which suggests a good discriminant validity of the PARIS.

**Figure 2:**
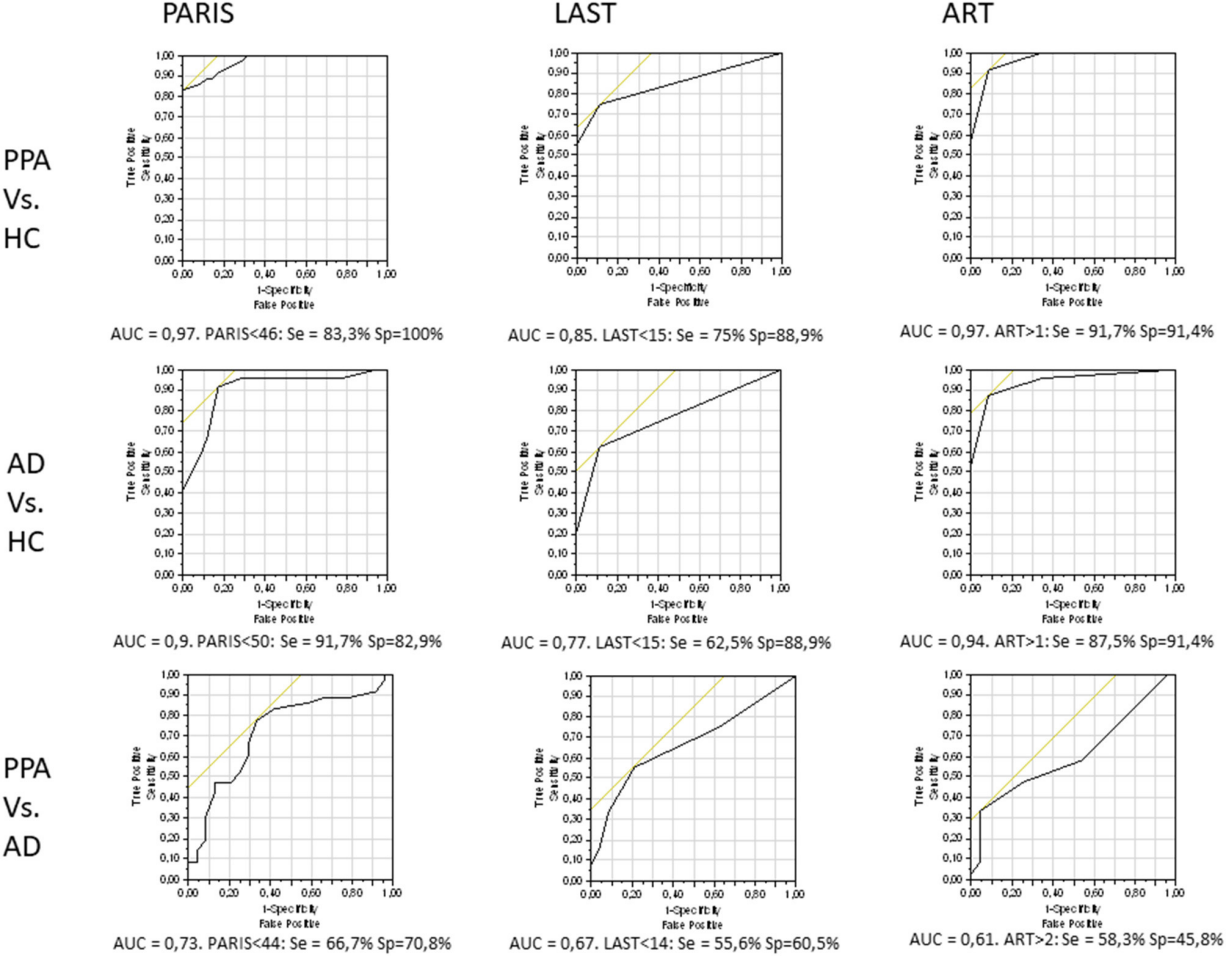
ROC curves, and sensitivity & specificity values regarding the best cut-off scores, for each test and all pair-wise group comparisons. HC, healthy controls.

Regarding the areas under the ROC curves (AUC), the analyses showed that both the PARIS and ART had good sensitivity/specificity for distinguishing PPA and AD patients from healthy controls (all AUCs ≥ 0.9). Regarding the distinction between PPA and AD patients the PARIS had the highest AUC. Specificities and sensitivities, and the best cut-off values of the PARIS, LAST, and ART for all group comparisons are shown in Figure 2.

### Follow-Up

We assessed the sensitivity of the three tests to identify language decline in the merged patient group including PPA and AD. Fifty participants were followed-up at 9 ± 1.2 months (15 healthy controls, 14 AD, 9 sv-PPA, 11 lv-PPA, 1 nfv-PPA). Forty-one participants withdrew from the study and four died. The sensitivity of the three tests to language decline as measured by SRM is illustrated in Table 4. The magnitude of score variation over time was higher in patients for the PARIS than for the LAST or ART. Patients lost 2.6 score-points (± 4.5) in the PARIS whereas they lost only 0.3 score-points (±1.3) in the LAST and 1.3 score-points (±2.8) in the ART. For healthy controls the score losses were similar in the three tests (PARIS: 0.5 score-points (±2.1), LAST: 0.1 score-points (±0.3), ART: 0.2 score-points (±0.9).

**Table 4:** Variation between baseline and the 9-month follow-up assessments for the three language tests.

	**Patients (*n* = 35)**			**Controls (*n* = 15)**		
	**Mean variation ± SD**	***p***	**SRM**	**Mean variation ± SD**	***p***	**SRM**
PARIS	2.6 ± 4.5	0.0020	0.565	−0.5 ±2.1	0.3963	0.226
LAST	0.3 ± 1.3	0.1404	0.263	0.1 ± 0.3	0.3343	0.258
ART	1.3 ± 2.8	0.0099	0.478	0.2 ± 0.9	0.3840	0.232

*SRM, Standardized Response Means (mean decline/standard deviation of decline)*.

## Discussion

We developed and evaluated a rapid language test (“PARIS”) conceived for PPA, and more generally for degenerative diseases affecting language, while comparing its outcomes in PPA variants and AD with two rapid language tools validated in post-stroke aphasia (“LAST,” “ART”). The PARIS was designed to qualify and quantify language disorders and decline by exploring five major domains of language (lexical, semantic, phonological, morpho-syntactic, verbal short-term memory capacities), and by using stimuli with graduated complexity regarding linguistic parameters to ensure its diagnostic sensitivity. The results show that the application duration of the PARIS is <11 min across all patients of our populations and that it demonstrates a high inter-examiner consistency. It reliably distinguishes between AD and PPA, allows for a rapid classification of the two presumably most frequent PPA variants (lv-PPA, sv-PPA) (24), and it sensitively detects language decline in neurodegenerative conditions after a time laps of 9 months. Moreover, the PARIS offers a good internal validity indicating the absence of redundant subtests. Likewise, despite the lack of a gold standard test for language disorders in PPA, the PARIS demonstrates a good external validity reflected by significant correlations between PARIS scores and scores of the DO80, subtest scores of the PARIS and the BDAE, as well as between PARIS, LAST and ART scores.

The diagnosis of PPA variants is a challenge for non-experts and experts reflected by a non-negligible proportion of so-called unclassifiable PPA patients (26, 27) who are not captured by the current diagnosis criteria (1). The diagnostic difficulty is partly related to the lack of a standardized test dedicated to PPA given that the numerous language tests, which are currently used have variable sensitivity for the main language features of PPA. Thus, some patients who might for example have lv-PPA are under-diagnosed because a mandatory criterion such as sentence repetition disorders is not detected when sentence length is not systematically increased. The same holds for word finding during picture naming or single-word comprehension capacities, which might be under-diagnosed because linguistic variables such as lexical frequency are not systematically varied. In the PARIS we therefore carefully graduated such language parameters to increase the sensitivity to PPA variant diagnosis. This approach allowed for a reliable classification of the two presumably most frequent PPA variants, namely lv-PPA and sv-PPA (24), which were initially included upon the diagnostic gold standard, i.e., clinical expertise in PPA (2). However, to validate the reliability of the PARIS for PPA diagnosis/classification future studies should apply it to a larger sample of patients, comprising more particularly a sufficient sample size of the rarest PPA variant, i.e., nfv-PPA, which has a low prevalence within the PPA spectrum as reflected by our study and reported by prior investigations (24). It is important to note that the proportional/relative frequency/prevalence of PPA variants is a ratio between the different variants once expert neurologists in tertiary memory/language expert centers have certified PPA variant diagnosis. This approach was applied in the investigation of Teichmann et al. (24). Intriguingly, two studies seem to indicate that nfv-PPA is a quite “frequent” PPA variant (28, 29) when adopting a purely epidemiologic approach in large populations of a given country/region. However, the accurate PPA variant diagnosis in these studies was not established in tertiary memory/language expert centers, which might induce important diagnosis/classification biases, and the relative/proportional frequency of the three main PPA variants was not provided given that the authors did not include lv-PPA. Hence, the issue of nfv-PPA should be cautiously addressed while taking into account that only patients with genuine impairment of syntactic and/or phonemic combinatorial capacities should be included in studies aiming at confirming the validation of the PARIS. Such studies should not include patients with exclusive motor speech impairment who do not correspond to the definition of aphasia but rather to the phenotypical/syndromic framework of “primary progressive apraxia of speech” (9, 10). Tools for identifying, quantifying and following-up this latter syndrome need to be developed in the future. Furthermore, the outcomes of the PARIS should also be confronted to test-independent variables such as imaging data providing characteristic patterns for each PPA variant (1, 30, 31). Finally, the PARIS needs to be evaluated and validated in other languages to become a useful tool beyond French speaking countries. Such a transposition to other languages, as for example English, will be easy to achieve given the rigorous linguistic-driven and well-defined test design. One should note that the PARIS is not thought to circumvent the various language batteries used in PPA and neurodegenerative conditions, but it might potentially represent an important adjunct or even lead to the validation of a pivotal test for PPA diagnosis, classification and follow-up.

Another major challenge regarding PPA diagnosis is the differentiation of PPA from the most frequent neurodegenerative condition comprising language disorders, namely AD. This distinction is crucial given that extra-linguistic cognitive features progressively emerge after some years of PPA evolution including memory disorders, especially in lv-PPA (32). Our findings show that the PARIS successfully separated AD from PPA patients who had a symptom evolution of 4 years (3-7) and 3 years (2–4.5), respectively, indicating that it might improve the reliability of PPA diagnosis in clinical settings of neurodegenerative diseases affecting cognitive abilities. Another quality of the PARIS is that making the distinction between AD and PPA patients is not restrained to experts of specialized cognitive centers given that the PARIS can be applied and exploited by non-expert practitioners (general practitioners, non-overspecialized neurologists) given the examiner-consistent and examiner-independent nature of the test. It should be noted that the diagnostic “PPA vs. AD” issue has previously been addressed in one study using the “Dépistage Cognitif de Québec” test (DCQ) suggesting that amnesic AD (and other degenerative neurocognitive diseases) can be differentiated from PPA (33). However, the DCQ is not centered on language but assesses various cognitive domains, is not designed for PPA given that some of PPA core features such as single-word comprehension are not assessed, is presumably not based on a robust binary rating scale (correct/incorrect) given that the authors did not mention the rating procedure, and consisted in a time-consuming administration duration (25 min in healthy controls, 40 min in patients). Hence, the PARIS appears to be the first rapid language-centered test reliably distinguishing PPA from AD, and providing a qualitative evaluation of impairment profiles and evolution in lv-PPA and sv-PPA.

A third challenge is the monitoring of language decline in PPA and more generally in neurodegenerative diseases affecting language capacities. There is currently no rapid language test which has been longitudinally applied to PPA and AD, and thus no reliable tool for the sensitive assessment of language decline in these diseases. However, the monitoring of language evolution is crucial to infer the severity of the degenerative process, to predict the functional evolution for individual patients, and to provide reliable endpoints in future therapeutic trials regarding PPA as well as AD. In previous trials the assessment of language performance and decline was severely limited given that the authors used language tests that target only a sole capacity, such as picture naming, or probing for rather general communication abilities (34). Our findings demonstrate that both the PARIS and the ART sensitively detect and quantify language decline at an interval of 9 months, but numerical values of sensitivity to decline were higher for the PARIS than for the ART. Regarding the three existing language tests that have been designed for PPA [PALS (2), PASS (3), SYDBAT (4)], their capacity to detect language decline was never longitudinally explored. Hence, it appears that the PARIS is the first rapid tool for the monitoring and quantification of language deterioration in neurodegenerative diseases, while enriching the range of existing rapid follow-up tests assessing the global decline of general cognitive capacities such as the MMSE (12). However, the sensitivity of the PARIS to language decline specifically in PPA needs to be evaluated and confirmed with a more important patient population given that the follow-up assessment in the present study was limited to 21 PPA patients.

Finally, regarding the three main language tests designed for and evaluated in PPA (PALS, PASS, SYDBAT), there are several advantages of the PARIS: it has a shorter application duration and the inter-examiner consistency was statistically analyzed, yielding an excellent score. Furthermore, in contrast to the three tests, the PARIS was evaluated longitudinally and demonstrated a good sensitivity to language decline, and it was applied to both PPA and AD showing its power to discriminate between these two major neurodegenerative conditions affecting language. Regarding the LAST and ART, which were designed for post-stroke aphasia, only the PARIS has the power to classify the two most frequent PPA variants, to distinguish between AD and PPA, and to detect language deterioration with a considerable sensitivity.

## Conclusion

The PARIS is a reliable, sensitive and examiner-independent test for PPA diagnosis and the classification of the two most frequent PPA variants. It constitutes a follow-up tool identifying the evolution velocity of language disorders in PPA and AD and can provide informative end-points in future therapeutic trials on PPA and other neurodegenerative language-destroying diseases.

However, one should keep in mind some limitations. First, our study included only few nfv-PPA patients because we cautiously excluded patients with pure speech apraxia taking into account criteria for PPA (1) and for “primary progressive apraxia of speech” (9, 10). To enlarge the population of nfv-PPA patients future studies need to include more important cohorts of such patients while distinguishing between populations with pure primary speech apraxia and with genuine primary non-fluent/agrammatic aphasia. Second, we evaluated the PARIS only in French and additional investigations should validate our findings using an English version. Such an English version has already been elaborated by our team and will be explored in English-speaking patients in the near future. A third limitation is that the discriminant validity was obtained by comparing the correlations between the three language tests (LAST, ART, and PARIS) to those of the PARIS and the MMSE or FAB. Although the correlations are numerically stronger between the language tests, the comparison with correlations of the PARIS with the MMSE or FAB is imperfect since, like most neuropsychological tests, the MMSE and the FAB are verbal tests that are necessarily altered in aphasic patients.

## Data Availability Statement

The raw data supporting the conclusions of this article will be made available by the authors, without undue reservation.

## Ethics Statement

The studies involving human participants were reviewed and approved by the study was approved by the local Ethical committee of the Pitié Salpêtrière Hospital. The patients/participants provided their written informed consent to participate in this study.

## Author Contributions

SE: study concept, patient recruitment, data analyses and interpretation of the results, writing the initial version of the manuscript. YS: patient recruitment, language assessment, revising the manuscript. CF and ER: study concept, revising the manuscript. SF, CA, and MN: language assessment, revising the manuscript. CA and BD: patient recruitment, revising the manuscript. ST: statistical analyses. MT: study concept, patient recruitment, data analyses and interpretation of the results, writing the final version of the manuscript.

## Conflict of Interest

The authors declare that the research was conducted in the absence of any commercial or financial relationships that could be construed as a potential conflict of interest.

## Supplementary Material

Supplementary Figure 1 Illustration of the PARIS (French version).

Supplementary Table 1 Comparison of the subtest scores of the PARIS, LAST and ART in sv-PPA and lv-PPA patients.
