# Supplementary figures and images for: A Reliable and Rapid Language Tool for the Diagnosis, Classification, and Follow-Up of Primary Progressive Aphasia Variants
Source: Front Neurol. 2021 Jan 5;11:571657. doi: 10.3389/fneur.2020.571657 (PMC7813774; doi:10.3389/fneur.2020.571657)

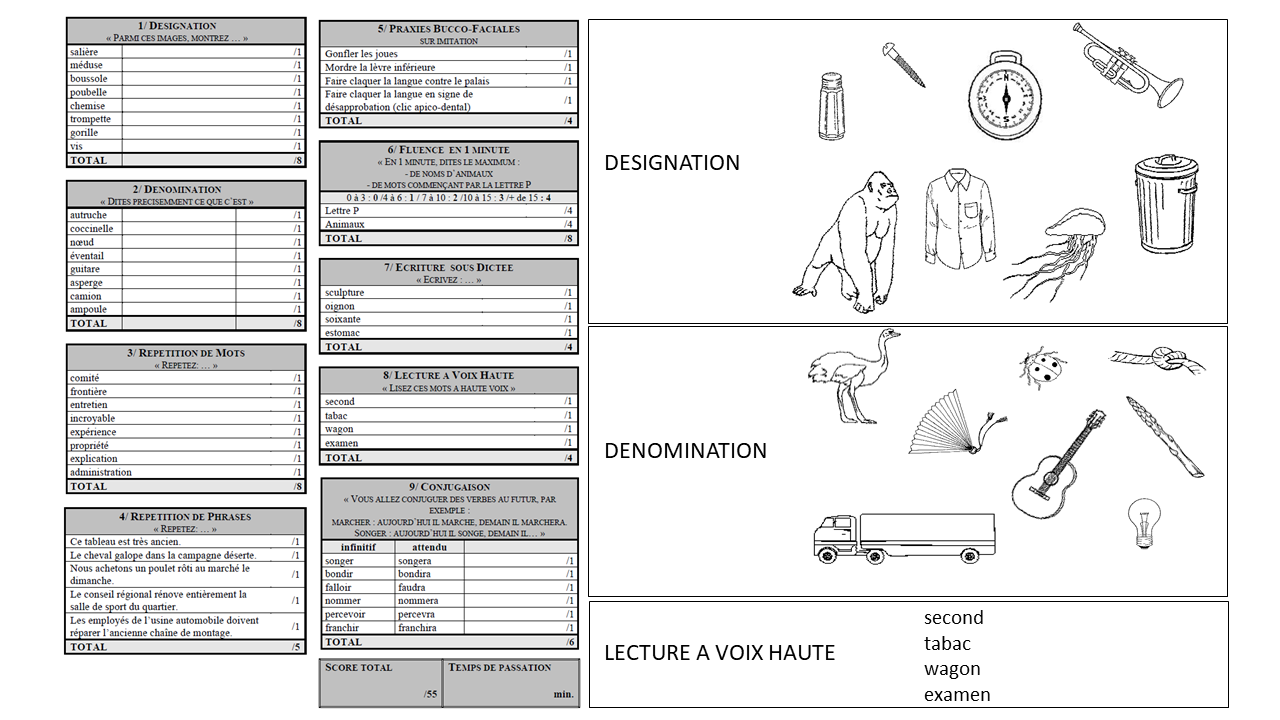

Supplement: Supplementary Figure 1 — Illustration of the PARIS (French version). [file Image_1.TIF]
