# Supplementary material for: A Reliable and Rapid Language Tool for the Diagnosis, Classification, and Follow-Up of Primary Progressive Aphasia Variants
Source: Front Neurol. 2021 Jan 5;11:571657. doi: 10.3389/fneur.2020.571657 (PMC7813774; doi:10.3389/fneur.2020.571657)
Supplement: Supplementary Table 1 — Comparison of the subtest scores of the PARIS, LAST and ART in sv-PPA and lv-PPA patients. [file Table_1.DOC]

**Supplementary Table**  Comparison of the subtest scores of the PARIS, LAST and ART in sv-PPA and lv-PPA patients.

|  | lvPPA | svPPA | P value* |
| --- | --- | --- | --- |
| **PARIS** | | | |
| Picture designation (/8) | 8 (8-8) | 7 (6-8) | 0,0001** |
| Picture naming (/8) | 5 (3,5-7) | 4 (3-5) | 0,22 |
| Word repetition (/8) | 8 (8-8) | 8 (8-8) | 0,35 |
| Sentence repetition (/5) | 1 (1-1,5) | 3 (3-3) | 0,0014** |
| Oro-facial praxis (/4) | 3 (3-4) | 4 (3-4) | 0,11 |
| 1 mn categorical (animals) and lexical (‘P’) fluencies (/8) | 3 (3-4,5) | 5 (4-6) | 0,06 |
| Irregular word reading (/4) | 2 (2-3) | 2 (2-4) | 0,86 |
| Irregular word writing (/4) | 4 (4-4) | 4 (4-4) | 0,58 |
| Verb conjugation (/6) | 5 (4-6) | 6 (5-6) | 0,13 |
| **LAST** | | | |
| Picture naming (/5) | 5 (4-5) | 5(3-5) | 0,3 |
| Word repetition (/2) | 1 (1-2) | 2 (2-2) | 0,003 |
| Counting from one to ten (/1) | 1 (1-1) | 1(1-1) | 0.4 |
| Picture designation (/4) | 4 (4-4) | 4 (4-4) | 0.25 |
| Order execution (/3) | 2 (2-3) | 3 (2-3) | 0.23 |
| **ART** | | | |
| Order execution (/5) | 0 (0-0,75) | 0 (0-0) | 0.45 |
| Word repetition (/6) | 0 (0-0) | 0 (0-0) | 1 |
| Sentence repetition (/2) | 0 (0-0) | 0 (0-0) | 1 |
| Picture naming (/6) | 1 (0-2) | 1 (0-2) | 0,91 |
| Dysarthria (/3) | 0 (0-1) | 0 (0-0) | 0,11 |
| 1 min categorical fluency (animals) (/4) | 2 (1,25 -3) | 2 (1-2,5) | 0,25 |

* Wilcoxon Test, ** significant after Bonferroni correction
